# The Ukraine crisis: Mental health resources for clinicians and researchers

**DOI:** 10.1002/jts.22837

**Published:** 2022-04-02

**Authors:** Mark Shevlin, Philip Hyland, Thanos Karatzias, Nino Makhashvili, Jana Javakhishvili, Bayard Roberts

**Affiliations:** ^1^ School of Psychology Ulster University Coleraine Northern Ireland; ^2^ Department of Psychology Maynooth University Maynooth Ireland; ^3^ School of Health and Social Care Edinburgh Napier University Edinburgh United Kingdom; ^4^ Global Initiative on Psychiatry Tbilisi Georgia; ^5^ School of Business, Technology, and Education Ilia State University Tbilisi Georgia; ^6^ Department of Health Services Research and Policy London School of Hygiene and Tropical Medicine London United Kingdom

## Abstract

The mental health consequences of the war in Ukraine will be enormous. Mental health professionals who are providing care for people in Ukraine, or those resettled elsewhere, may require access to standardized and validated assessment tools. We have developed a repository of mental health measures that are available in Ukrainian, Russian, and English and can be accessed at http://www.traumameasuresglobal.com/ukraine.

The world has watched in horror as the recent events in Ukraine have unfolded. After a long period of experiencing anticipatory threat as Russian troops amassed on their border, the Ukrainian population is now subjected to a full military invasion, with death, destruction, and displacement a daily reality. It almost goes without saying that the mental health consequences for the Ukrainian people will be enormous. In 2016, there were already 1,800,000 internally displaced persons (IDPs) in Ukraine (United Nations High Commissioner for Refugees, 2016), and the Internally Displaced Persons Mental Health Survey in Ukraine (Roberts et al., 2017), carried out from March to May 2016, demonstrated that mental health problems were common among Ukrainian IDPs, with 22% of respondents reporting symptoms of depression, 18% reporting symptoms of anxiety (Roberts et al., 2019), and 55% endorsing significant levels of somatization (Cheung et al., 2018). Among current drinkers, 14.3% of men and 1.7% of women reported potentially hazardous drinking (Ramachandran et al., 2019). Moreover, the prevalence of PTSD, as assessed using the *International Classification of Diseases* (11th rev.; *ICD‐11*; World Health Organization [WHO], 2019) criteria, was 21% in the total sample and 57.6% among individuals experiencing clinically significant levels of impairment (Shevlin et al., 2018). To place all this in a global context, a recent study estimated the cross‐national lifetime prevalence of PTSD to be 3.9% (Koenen et al., 2017). These mental health needs among IDPs in Ukraine occurred in the context of very limited mental health service availability, with 74% of participants who likely required mental health and psychosocial support care not receiving these services (Roberts et al., 2019).

At present, the Ukrainian community of mental health professionals is providing help and support in unimaginably difficult conditions; several hotlines operate, volunteers are mobilized and trained in psychological first aid provision, and mobile crisis intervention stations are in operation. However, in chaotic situations, there is a risk of offering non–evidence‐based interventions, and the collection of evidence is crucially important and needs to be considered as an inevitable part of ongoing and future interventions. Resources are required for this.

The International Trauma Consortium (ITC) is a collaboration of researchers and clinicians working in the field of traumatic stress studies. To assist mental health clinicians and researchers, we have collated and hosted a range of established and validated mental health measures that have been translated into Ukrainian and Russian; these are now freely available to download at https://www.traumameasuresglobal.com/

The measures are:

1. The International Trauma Questionnaire (ITQ; Cloitre et al., 2018), a measure of *ICD‐11* posttraumatic stress disorder (PTSD) and complex PTSD
2. The Patient Health Questionnaire–9 (PHQ‐9; Kroenke et al., 2001), a brief self‐report measure of depressive symptoms
3. The Generalized Anxiety Disorder–7 (GAD‐7; Spitzer et al., 2006), a brief self‐report measure of generalized anxiety
4. The Life Events Checklist for DSM‐5 (LEC‐5; Gray et al., 2004), a measure of trauma exposure
5. The Patient Health Questionnaire–15 (Kroenke et al., 2002), a measure of somatic symptom severity
6. The World Health Organization Disability Assessment Schedule–II (WHODAS‐II; WHO, 2001), a brief measure of functional impairment

We hope that these measures are helpful to clinicians and researchers who will be dealing with the psychological aftermath of the conflict in Ukraine. We intend to continue to expand this repository—for example, child and adolescent measures will soon be added—and we would welcome any other resources (email Mark Shevlin or Philip Hyland).
